# Nurse-Teachers for Invalid Children

**Published:** 1920-12-04

**Authors:** 


### Nurse-Teachers for Invalid Children

The problem of educating children of school age who for any reason are under prolonged treatment in institutions has been exercising the attention of the education authorities for some time past, but is still unsolved. Not long ago Mr. Herbert Nield asked the Minister of Health in the House of Commons whether he was aware that at the Isleworth Hospital, under the Brentford Guardians, there are always a number of children suffering from various complaints which debar them from being educated, and whether he will take steps to provide a nurse-teacher for such children while they are in the wards. Dr. Addison could not hold out any hope of a grant being made by the Ministry of Health for this purpose, though he cordially agreed it was desirable such a teacher should be appointed. In this country we are far behind our Canadian cousins in this respect. The fifth annual Report of the Ontario Inspector of Auxiliary Classes, which we have lately received, shows that a vigorous system is in force for educating, even in remote places, such children as are prevented by physical or mental defects from attendance at school. At the Hospital for Sick Children in Toronto a head-teacher and assistant attend daily for the instruction of some forty little patients, and at the Home for Incurable Children, in the same city, there is an afternoon class of eighteen children, under a trained teacher, which has proved a great help and source of hope and cheerfulness. Pending the development of such work, under trained and salaried teachers, in this country, we believe much more might be done on a voluntary basis by Red Cross workers to interest and instruct children under treatment for long periods.

